# *De novo* genome sequencing and comparative analyses of the clinically relevant species *Mucor ardhlaengiktus, Mucor circinelloides, Mucor griseocyanus*, and *Mucor janssenii*

**DOI:** 10.1093/mmy/myaf081

**Published:** 2025-09-05

**Authors:** Bert Gerrits van den Ende, Michaela Lackner, Ferry Hagen

**Affiliations:** Westerdijk Fungal Biodiversity Institute, Utrecht, The Netherlands; Institute for Hygiene and Medical Microbiology, Medical University of Innsbruck, Innsbruck, Austria; Westerdijk Fungal Biodiversity Institute, Utrecht, The Netherlands; Institute for Biodiversity and Ecosystem Dynamics, University of Amsterdam, Amsterdam, The Netherlands; Department of Medical Microbiology, University Medical Center Utrecht, Utrecht, The Netherlands

**Keywords:** *Mucorales*, *Mucoromycotina*, mitochondrial genome, nanopore sequencing, diagnostics

## Abstract

Mucormycosis is predominantly caused by members of the genera *Lichtheima, Mucor*, and *Rhizopus*. Here, we report the genome assemblies and comparative analyses of the clinically relevant species *Mucor ardhlaengiktus* (CBS 210.80), *Mucor circinelloides* (CBS 195.68), *Mucor janssenii* (CBS 205.68), and *Mucor griseocyanus* (CBS 116.08) to enable molecular analyses.

Mucormycosis is caused by a diverse group of early diverging fungi associated with the subphylum *Mucoromycota*. To date, 39 species dispersed over 11 genera have been reported to be of clinical significance.^1^ The most encountered species in a clinical setting belong to the genera *Lichtheimia, Mucor*, and *Rhizopus*, although with varying incidence in different geographic regions.^2^*Mucor* is with 13 species causing infections in humans the most relevant genus, but only *Mucor circinelloides* was so far found in nosocomial outbreaks.^3^ To date, only one clinically relevant representative (*M. lusitaniae*, NCBI accession number GCA_040256725.1) has been reported to have a chromosome-level assembly with 12 fragments, while other reported *de novo* assembled *Mucor* genomes have hundreds to thousands of contigs due to the use of short-read next-generation sequencing technologies.^4^ Here, we aim to provide high-quality genomes of the four clinically relevant *Mucor* species *M. ardhlaengiktus, M. circinelloides, M. griseocyanus*, and *M. janssenii* to enable molecular analyses as well as to use these *de novo* assembled genomes as reference genomes for outbreak-related analyses, such as variant calling.

The type strains of *M. ardhlaengiktus* (CBS 210.80^T^), *M. circinelloides* (CBS 195.68^NT^), *M. griseocyanus* (CBS 116.08^T^), and *M. janssenii* (CBS 205.68^T^) were subcultured in Malt Extract Agar (MEA; Oxoid, Basingstoke, UK) for 72 h at 35°C. Fungal cell material was collected to obtain genomic DNA according to a previously established cetyltrimethylammonium bromide extraction protocol.^5^ The quality and quantity of genomic DNA was assessed by the Qubit High Sensitivity Kit (ThermoFisher, Waltham, MA, USA) and 2% agarose-gel electrophoresis. Per strain, an input of 1 µg was used in order to prepare the library using the native ligation kit SQK-LSK109 in combination with EXP-NBD104 (ONT, Oxford Nanopore Technologies, Oxford, UK). The library was loaded onto a FLO-MIN106D R9.4.1 flow cell and raw data were collected into FAST5 files. Raw data were re-analyzed with MinKNOW v24.11.8 using the built-in dorado basecaller in super-accuracy mode with the model dna_r9.4.1_e8_sup@v3.3 (ONT). Per strain, data were collected into a single FASTQ file and subsequently processed using Chopper v0.8.0.^6^ Reads with a length of >1100 bp and a Q-score of ≥10 were selected and 50 bp of the head and tail were removed. NanoComp v1.12.0 was used to compare pre- and post-quality analyses, and to perform strain-to-strain analyses (Table 1).^6^*De novo* genome assemblies were performed using Flye v2.9.5-b1801 (Table 1).^7^ The genomes of *M. circinelloides, M. griseocyanus*, and *M. janssenii* were found to be 37 792 404, 36 594 021, and 38 038 536 bp in size, respectively. This is comparable to the reported genome sizes of *M. hiemalis* (32 290 736 bp), *M. irregularis* (33 988 018 bp), and 30 *M. circinelloides* strains (34 510 016 bp ± 308 217 bp), but smaller than several reported genomes of *Mucor* species (range 35.00–46.92 Mbp).^4,8,9^ The genome of *M. ardhlaengiktus* with a size of 51 526 660 bp was significantly larger (37%) than the other analyzed *Mucor* genomes.

**Table 1.** Genome assembly information of the four sequenced *Mucor* type strains.

	*Mucor ardhlaengiktus* CBS 210.80	*Mucor circinelloides* CBS 195.68	*Mucor griseocyanus* CBS 116.08	*Mucor janssenii* CBS 205.68
***Sequence read statistics***				
Mean read length raw/filtered (bp)	2358.7/5 773.9	1492.5/3 888.3	939.3/3 173.2	930.3/3 237.2
Mean read quality raw/filtered	12.4/12.4	12.6/12.6	13.2/13.1	13.2/13.1
Median read length raw/filtered (bp)	602/2 957	552/2 093	390/1 979	368/2 015
Median read quality raw/filtered	12.6/12.5	12.8/12.7	13.5/13.5	13.6/13.5
Number of reads raw/filtered (bp)	1 135 718/404 666	1 758 075/521 410	2 204 961/419 239	2 516 333/472 081
Read length N50 raw/filtered (bp)	8789/10 714	4133/6 382	2186/4 324	2307/4 402
SD read length raw/filtered (bp)	5185.6/7490.2	3144.4/4929.5	1884.1/3417.8	1944.8/3572.4
Total bases raw/filtered (bp)	2 678 785 588/2 336 506 905	2 623 911 194/2 027 407 212	2 071 049 576/1 330 339 707	2 340 837 505/1 528 219 173
Number of reads >Q10 raw/filtered	1 112 109/403 972	1 727 523/520 502	2 166 051/418 554	2 469 338/471 281
Base pairs >Q10 raw/filtered (Mbp)	2326.4/1526.6	2611.6/2025.4	2058.3/1329.0	2669.4/2334.7
Number of reads >Q15 raw/filtered	121 409/20 384	245 805/46 338	607 831/96 485	716 138/109 708
Base pairs >Q15 raw/filtered (Mbp)	541.2/322.6	191.9/111.1	473.6/279.5	109.8/75.7
Number of reads >Q20 raw/filtered	4111/5	7258/106	30 332/70	41 844/120
Base pairs >Q20 raw/filtered (Mbp)	6.6/0.2	1.4/0.1	5.1/0.1	0.5/0.0
Number of reads >Q25 raw/filtered	409/0	615/0	3 118/0	4 898/0
Base pairs >Q25 raw/filtered (Mbp)	0.2/0.0	0.0/0.0	0.2/0.0	0.0/0.0
Number of reads >Q30 raw/filtered	6/0	9/0	33/0	74/0
Base pairs >Q30 raw/filtered (Mbp)	0.0/0.0	0.0/0.0	0.0/0.0	0.0/0.0
***Genome statistics***				
Total read length (bp)	2 336 506 905	2 027 407 212	1 330 339 707	1 528 219 173
Reads N50/N90 (bp)	10 714/2214	6382/1497	4324/1384	4402/1407
Total genome size (bp)	51 526 660	37 792 404	36 594 021	38 038 536
GC%	37.03%	39.43%	40.48%	40.34%
Fragments	34	27	45	78
Fragments N50 (bp)	2 706 610	2 387 538	1 729 767	926 921
Largest fragment (bp)	3 998 929	4 948 823	3 582 357	1 930 117
Mean coverage	44X	50X	35X	39X
Mitochondrial genome size (bp)	34 011	30 803	32 283	32 525
Mitochondrial genome coverage	2072X	2540X	1021X	1291X
Predicted genes	22 373	14 360	13 926	12 734
***BUSCO analysis using the mucoromycota_odb10 database (1 614 BUSCOs included)***				
Single BUSCOs	1458 (90.33%)	1611 (99.81%)	1613 (99.94%)	1601 (99.19%)
Duplicated BUSCOs	151 (9.36%)	3 (0.19%)	0 (0.0%)	5 (0.31%)
Fragmented BUSCOs	0 (0.0%)	0 (0.0%)	0 (0.0%)	2 (0.12%)
Missing BUSCOs	5 (0.31%)	0 (0.0%)	1 (0.06%)	6 (0.37%)
***BUSCO analysis using the mucorales_odb10 database (2 449 BUSCOs included)***				
Single BUSCOs	2214 (90.33%)	2441 (99.67%)	2444 (99.8%)	2431 (99.27%)
Duplicated BUSCOs	226 (9.23%)	8 (0.33%)	5 (0.2%)	9 (0.37%)
Fragmented BUSCOs	0 (0.0%)	0 (0.0%)	3 (0.12%)	0 (0.0%)
Missing BUSCOs	9 (0.37%)	0 (0.0%)	6 (0.24%)	0 (0.0%)
***Data availability via NCBI BioProject PRJNA1221132***				
NCBI BioSample	SAMN46730506	SAMN46730504	SAMN46730503	SAMN46730505
NCBI Genome	JBLNHJ000000000	JBLNHH000000000	JBLNHG000000000	JBLNHI000000000

The circular mitochondrial genomes of the four *Mucor* species were between 30 803 and 34 011 bp in size (Table 1); this is close to the genome size of *M. piriformis* (33 530 bp), which was classically determined, and to that of *M. velutinosus* (39 823 bp).^10,11^ However, an environmental strain of *M. indicus* had a reported mitochondrial genome size of 61 400 bp.^12^

The GC content of the *Mucor* genomes reported here was 37.03%–40.48%, similar to the 32%–42% calculated previously for 11 *Mucor* species (Table 1).^8,11^

To assess the genome quality, we applied compleasm v0.2.6 to search for the presence of single-copy orthologs for which the ‘Benchmarking Universal Single-Copy Orthologs’ (BUSCO) databases mucorales_odb10 and mucoromycota_odb10 were used (Table 1).^13^ The *de novo* assembled genomes of *M. circinelloides, M. griseocyanus*, and *M. janssenii* were found to be highly complete with BUSCO scores of >99.2% for both BUSCO databases. However, the genome of *M. ardhlaengiktus* was found to contain >9% of duplicated BUSCOs, which might suggest that—given the large genome size of 51 Mbp—the sequenced strain is an aneuploid hybrid.

Helixer v0.3.4 was run to determine the number of genes, with 12 734 predicted genes for *M. griseocyanus*, 13 296 for *M. circinelloides*, and 14 360 for *M. janssenii*.^14^ Strikingly, 22 373 genes were predicted in the genome of *M. ardhlaengiktus* (Table 1).

To calculate the average nucleotide identity (ANI) between the four genomes, we used OrthoANI v0.6.0.^15^ This resulted in ANI values of 71.37%–71.62% between *M. ardhlaengiktus* and the other three *Mucor* strains, and the ANI between *M. circinelloides* vs. *M. griseocyanus* and *M. janssenii* was 82.69% and 82.35%, respectively. The ANI value between *M. griseocyanus* and *M. janssenii* was 85.87%. An ANI value below 95% supports species delination for bacteria; however, this is yet to be determined for fungi.^16^ The low ANI value for *M. ardhlaengiktus* seems to be due to the extensive difference in genome size from the other *Mucor* strains (Table 1).

The FASTQ data and genome assemblies have been deposited in the NCBI databases (Table 1). The strains are available via the CBS Culture Collection hosted at the Westerdijk Fungal Biodiversity Institute, Utrecht, The Netherlands.

## References

[1] Walther G, Wagner L, Kurzai O. Updates on the taxonomy of *Mucorales* with an emphasis on clinically important taxa. J Fungi (Basel). 2019; 5(4): 106. 31739583 10.3390/jof5040106 PMC6958464

[2] Skiada A, Pavleas I, Drogari-Apiranthitou M. Epidemiological trends of mucormycosis in Europe, comparison with other continents. Mycopathologia. 2024; 189(6): 100. 39565510 10.1007/s11046-024-00907-5

[3] Walther G, Wagner L, Kurzai O. Outbreaks of *Mucorales* and the species involved. Mycopathologia. 2020; 185(5): 765–781. 31734800 10.1007/s11046-019-00403-1

[4] Garcia-Hermoso D, Criscuolo A, Lee SC et al. Outbreak of invasive wound mucormycosis in a burn unit due to multiple strains of *Mucor circinelloides* f. *circinelloides* resolved by whole-genome sequencing. mBio. 2018; 9(2): e00573–18. 29691339 10.1128/mBio.00573-18 PMC5915733

[5] Navarro-Muñoz JC, de Jong AW, Gerrits van den Ende B et al. The high-quality complete genome sequence of the opportunistic fungal pathogen *Candida vulturna* CBS 14366(T). Mycopathologia. 2019; 184(6): 731–734. 31734799 10.1007/s11046-019-00404-0

[6] De Coster W, Rademakers R. NanoPack2: population-scale evaluation of long-read sequencing data. Bioinformatics. 2023; 39(5): btad311. 37171891 10.1093/bioinformatics/btad311 PMC10196664

[7] Kolmogorov M, Yuan J, Lin Y, Pevzner PA. Assembly of long, error-prone reads using repeat graphs. Nat Biotechnol. 2019; 37(5): 540–546. 30936562 10.1038/s41587-019-0072-8

[8] Lebreton A, Corre E, Jany JL et al. Comparative genomics applied to *Mucor* species with different lifestyles. BMC Genomics. 2020; 21(1): 135. 32039703 10.1186/s12864-019-6256-2 PMC7011435

[9] Zhang M, Xu W, Mei H et al. Comparative genomics predict specific genes in potential *Mucorales* identification. Arch Microbiol. 2023; 205(9): 320. 37640972 10.1007/s00203-023-03659-w

[10] Papp T, Palágyi Z, Ferenczy L, Vágvölgyi C. The mitochondrial genome of *Mucor piriformis*. FEMS Microbiol Lett. 1999; 171(1): 67–72. 9987843 10.1111/j.1574-6968.1999.tb13413.x

[11] Conlan S, Subramanian P, Plata Barril B et al. Whole-genome sequence of *Mucor velutinosus* NIH1002, a strain associated with disseminated disease. Microbiol Resour Announc. 2024; 13(7): e0006224. 38899875 10.1128/mra.00062-24 PMC11256851

[12] Deng Y, Chen G, Bao X, He J, Li Q. Characterization of the complete mitochondrial genome of *Mucor indicus* Lendn. 1930 (Mucorales: Mucoraceae), isolated from the wine fermentation system. Mitochondrial DNA B Resour. 2024;9(6): 845–849. 38939449 10.1080/23802359.2024.2371376 PMC11210418

[13] Huang N, Li H. compleasm: a faster and more accurate reimplementation of BUSCO. Bioinformatics. 2023; 39(10): btad595. 37758247 10.1093/bioinformatics/btad595 PMC10558035

[14] Stiehler F, Steinborn M, Scholz S, Dey D, Weber APM, Denton AK. Helixer: cross-species gene annotation of large eukaryotic genomes using deep learning. Bioinformatics. 2021; 36(22–23): 5291–5298. 33325516 10.1093/bioinformatics/btaa1044 PMC8016489

[15] Lee I, Ouk Kim Y, Park SC, Chun J. OrthoANI: an improved algorithm and software for calculating average nucleotide identity. Int J Syst Evol Microbiol. 2016; 66(2): 1100–1103. 26585518 10.1099/ijsem.0.000760

[16] Goris J, Konstantinidis KT, Klappenbach JA, Coenye T, Vandamme P, Tiedje JM. DNA–DNA hybridization values and their relationship to whole-genome sequence similarities. Int J Syst Evol Microbiol. 2007; 57(Pt 1): 81–91. 17220447 10.1099/ijs.0.64483-0

